# Performance in the 6-minute walk test and postoperative pulmonary complications in pulmonary surgery: an observational study

**DOI:** 10.1590/bjpt-rbf.2014.0119

**Published:** 2016-01-19

**Authors:** Bruna F. A. Santos, Hugo C. D. Souza, Aline P. B. Miranda, Federico G. Cipriano, Ada C. Gastaldi

**Affiliations:** 1 Curso de Fisioterapia, Departamento de Biomecânica, Medicina e Reabilitação do Aparelho Locomotor, Faculdade de Medicina de Ribeirão Preto (FMRP), Universidade de São Paulo (USP), Ribeirão Preto, SP, Brazil; 2 Programa de Pós-graduação em Reabilitação e Desempenho Funcional, Departamento de Biomecânica, Medicina e Reabilitação do Aparelho Locomotor, Faculdade de Medicina de Ribeirão Preto (FMRP), USP, Ribeirão Preto, SP, Brazil; 3 Departamento de Clínica Cirúrgica, Faculdade de Medicina de Ribeirão Preto (FMRP), USP, Ribeirão Preto, SP, Brazil

**Keywords:** functional capacity, six-minute walk test (6MWT), thoracotomy, pulmonary complications, physical therapy

## Abstract

**OBJECTIVES:**

To assess functional capacity in the preoperative phase of pulmonary surgery by comparing predicted and obtained values for the six-minute walk test (6MWT) in patients with and without postoperative pulmonary complication (PPC)

**METHOD:**

Twenty-one patients in the preoperative phase of open thoracotomy were evaluated using the 6MWT, followed by monitoring of the postoperative evolution of each participant who underwent the routine treatment. Participants were then divided into two groups: the group with PPC and the group without PPC. The results were also compared with the predicted values using reference equations for the 6MWT

**RESULTS:**

Over half (57.14%) of patients developed PPC. The 6MWT was associated with the odds for PPC (odds ratio=22, p=0.01); the group without PPC in the postoperative period walked 422.38 (SD=72.18) meters during the 6MWT, while the group with PPC walked an average of 340.89 (SD=100.93) meters (p=0.02). The distance traveled by the group without PPC was 80% of the predicted value, whereas the group with PPC averaged less than 70% (p=0.03), with more appropriate predicted values for the reference equations

**CONCLUSIONS:**

The 6MWT is an easy, safe, and feasible test for routine preoperative evaluation in pulmonary surgery and may indicate patients with a higher chance of developing PPC.

## Introduction

Postoperative pulmonary complications (PPCs) are common and are a major cause of morbidity and mortality^1^. They are defined as complications that present within 30 days after the surgical procedure and include mechanical ventilation for more than 24 hours, hypoxemia, atelectasis, hemoptysis, empyema, and death caused by heart or respiratory failure^2^.

The preoperative evaluation is a fundamental step in identifying risk factors for the development of these complications, which can be related to the patient and to the planned surgical procedure. Tests such as spirometry and arterial blood gases are generally used in the preoperative evaluation of all patients eligible for lung resection, in order to assess the risk of complications and calculate the residual forced expiratory volume in one second (FEV_1_) to determine surgical indication. Although FEV_1_ obtained by spirometry has been widely used for this risk stratification process, cases of increased risk may require additional tests, such as lung diffusion testing (DLCO), and methods to estimate residual postoperative lung function, such as scintigraphy and cardiopulmonary exercise tests^1^^,^ 3.

Cardiopulmonary exercise testing is considered extremely important in identifying patients at greater risk of complications and mortality, and according to the European functional assessment algorithm, should be performed especially when the predicted FEV_1_ or DLCO are below 80%. However, its application is restricted due to the limited availability of the test, which has stimulated the search for methods to provide similar information that is also simpler and more cost-effective^3^.

Among the alternatives, the step test, the shuttle walk test, and the six-minute walk test (6MWT) have been studied^1^^,^ 3. Despite the satisfactory results found in the performance of these tests, some points related to the methods - especially the control of exercise intensity, the variability of the results, and level of evidence - require a standardization and interpretation of the results obtained by patients^3^.

The 6MWT is simple to perform, practical, and does not require special equipment or facilities. The 6MWT is intended to assess, at submaximal levels, exercise walking for six minutes. The test provides an assessment of all systems involved during exercise, including the cardiopulmonary system and the peripheral muscles. As walking is a routine activity and the intensity is defined by the patient, it is usually well tolerated and can be used easily in the preoperative period^4^.

The 6MWT follows a standardization proposed by the American Thoracic Society (ATS)^4^, which makes it a safer and more reproducible technique, however patients may vary the intensity of exercise, which can lead to different results, even when compared with predicted values, which can be predicted by several reference equations^5^^-^ 11. Additionally, since the predicted values were established for the general population, there is no information available about the expected performance of patients in the preoperative phase, which may be compromised by the underlying disease.

The aim of this study was to optimize the care requirements of the population submitted to lung surgery via thoracotomy by evaluating functional capacity prior to surgery using the 6MWT and the predicted values obtained in the reference equations in patients with and without PPC.

## Method

This is a prospective observational study, conducted in the years 2012 to 2014, with patients from the Clinical Hospital of Ribeirão Preto Medical School, Universidade de São Paulo (HC-FMRP-USP), Ribeirão Preto, SP, Brazil.

**Figure 1:**
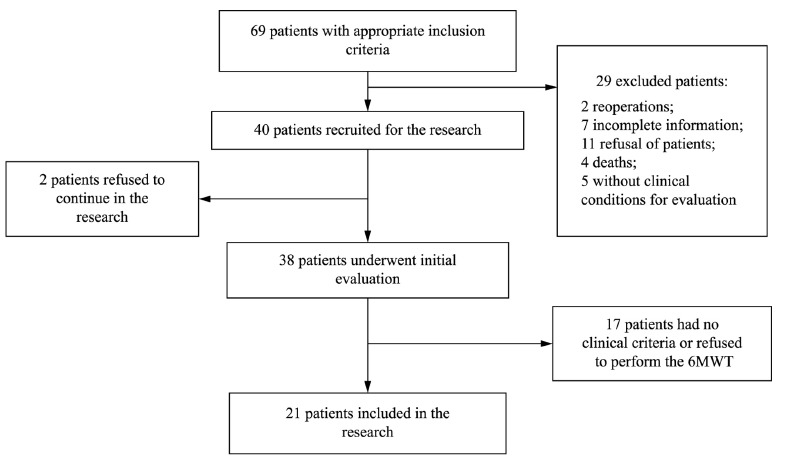
- Study flowchart.

For this research, we initially recruited 69 patients, all with surgical indication of open thoracotomy with or without pulmonary resection, however only 21 were able to participate, as shown in Figure 1.

Patients were over 18 years old, with the ability to walk, without cognitive impairment, clinically stable, and agreed to participate and signed an informed consent form. The exclusion criteria were as follows: orthopedic disorders; balance disorders; inability to understand commands for testing; or uncontrolled pain.

The evaluation was performed in the preoperative period and included the patient's history, physical examination, and the 6MWT. Before and after the 6MWT, the following measurements were taken: blood pressure; pulse oximetry; heart rate; visual analogue scale (VAS) for pain; and dyspnea level through the Borg scale. In cases of oxygen saturation lower than 85%, the test was interrupted and restarted after the oxygen was supplemented. The 6MWT was performed in a corridor 30 meters long, following the standard established by the American Thoracic Society^4^, using a stopwatch, two cones for delimitation of the circuit, one sphygmomanometer (Premium^(r)^ ESFHS50 model, China), one stethoscope (adult BD^(r)^MDF727, United States), and an oximeter (Moriya^(r)^ MD300C1, China).

During the postoperative hospital stay, patients received routine clinical care and physical therapy. During this period and after hospital discharge, the PPCs developed by patients were recorded. For data analysis, patients were divided into two groups according to postoperative outcome. The first group included the patients who did not develop PPCs, called the group without PPC, and the second group included the patients who developed PPCs, called the group with PPC.

PPCs were considered those complications presented by patients after surgery, such as mechanical ventilation for more than 24 hours, hypoxemia, atelectasis, hemoptysis, empyema, and death caused by heart or respiratory failure^2^.

The results were also compared with different equations to predicted values of the 6MWT^5^^-^ 11. The equations used were Soares and Pereira^8^, Britto et al.^11^ (A = model considering heart rate; B = model not considering the heart rate), Dourado et al.^10^, Iwama et al.^7^, Gibbons et al.^6^, and Enright and Sherrill^5^, selected because they were taken in groups of the same age group.

The normal distribution of data was assessed using the Shapiro-Wilk test and the results were expressed as mean and standard deviation. The comparison between the groups with and without PPC was made with the application of the unpaired t-test, and the comparison between the values obtained and provided by the various reference equations was performed with one-way ANOVA followed by the Tukey test for multiple comparisons, using Prism software 5^(r)^, with a p-value of 0.05.

To examine a possible association with anthropometric data, 6MWT, and FEV_1_(independent variables) with the risk of PPC (dependent variable), the method of simple and multiple logistic regression was used, and the results were presented in odds ratio (OR) using PROC LOGISTIC of SAS^(r)^ 9.0 software.

The design of this study was approved by the Research Ethics Committee of HCRP and FMRP, Universidade de São Paulo (USP), Ribeirão Preto, SP, Brazil, 043627/2012 process.

## Results

Functional capacity was assessed by the 6MWT in 21 patients, 11 females (52.11%) and 10 males (47.61%) in the preoperative period of open thoracotomy. Patients walked an average distance of 375.81 (SD=92.92) meters and had 7.71 (SD=3.10) days of hospital stay. Among these 21 patients, 12 went on to develop PPC (57.14% of participants) after surgery, while 9 evolved without PPC (42.86% of the participants).

The group without PPC had a mean age of 59.22 (SD=8.24) years, comprised 33.33% women and remained 6.11 (SD=1.26) days in hospital after surgery. The surgical procedures were pneumonectomy (1), lobectomy (2), segmentectomy (3), nodulectomy (2), and biopsy (1). The group with CPP had a mean age of 61.41 (SD=7.93) years, comprised 41.66% women and remained 8.91 (SD=3.55) days in hospital after surgery. The surgical procedures were pneumonectomy (1), lobectomy (4), segmentectomy (5), nodulectomy (1), and biopsy (1). The comparison of age, weight, height, and body mass index (BMI) of the two groups showed no statistically significant differences, but there was a difference for the expected percentage of FEV_1_ (p=0.04) (Table 1).

**Table 1:** - Patient characterization and 6MWT in the groups with and without PPC, expressed as a mean and standard deviation.

	Group without PPC	Group with PPC	P value
Age (years)	59.22 (8.24)	61.41 (9.93)	0.78
Gender	6F/3M	5F/7M	-
Weight (Kg)	68.42 (10.49)	73.25 (17.18)	0.69
Height (cm)	160.44 (7.55)	163.25 (10.01)	0.71
BMI (Kg/m^2^)	26.70 (4.01)	27.39 (5.17)	0.86
FEV_1_ (% of pred)	67.3 (12.8)	82.4 (21.8)	0.04
6MWD obtained (meters)	422.38 (2.18)	340.89 (100.93)	0.02

PPC=postoperative pulmonary complications; F=female; M=male; Kg=kilogram; cm=centimeters; BMI=body mass index; FEV1=forced expiratory volume in one second; pred=predicted; 6MWT=six-minute walk test.

**Figure 2:**
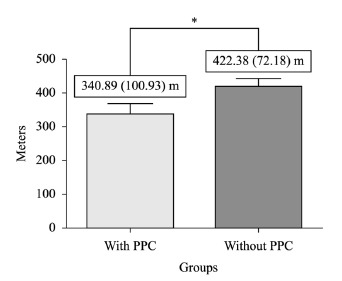
- Distance obtained during the 6MWT in patients with and without PPC. *p=0.02; 6MWD=six-minute walk distance; PPC= postoperative pulmonary complications

The group of patients without PPC walked an average of 422.38 (SD=72.18) meters, while the group with PPC walked an average of 340.89 (SD=100.93) meters. Statistical analysis showed that when comparing the 6MWT, the group without PPC traveled a greater distance than the group with PPC (p=0.02) (Tables 1 and 2 and Figure 2), with an estimated power of 95%.

In the group without PPC, the values were not different from those predicted by the equations. In this group, the percentage of predicted value ranged 66 83% of the prediction. However, in the group with PPC, the values were different from those predicted by the equations, ranging from 51-69% (p=0.03), except for the values predicted by Gibbons et al.^6^ (p=0.09) (Table 2). When the percentages of predicted value by different equations were compared in the groups with and without PPC, the percentage predicted by Gibbons et al.^6^ was different from those provided by all other equations (p=0.02) (Table 2).

**Table 2:** - 6MWT values obtained and percentage of the predicted values by different equations in groups with and without PPC.

		Without PPC			With PPC	
	Mean (SD)	CI95%	Obtained vs. pred	Mean (SD)	CI95%	Obtained vs. pred
Obtained (m)	422.38 (72.19)	366.89-477.87	P value	340.89 (100.93)	276.76-405.02	P value
Soares and Pereira^8^ (%)	81.25 (16.71)	68.41-94.09	0.22	65.06 (16.95)	54.29-75.83	0.04
Britto et al.^11^ A (%)	79.36 (16.77)	76.46-92.23	0.22	62.47 (16.44)	52.02-72.92	0.03
Britto et al.^11^ B (%)	79.80 (16.17)	67.37-92.22	0.22	62.84 (17.95)	51.44-74.24	0.03
Dourado et al.^10^ (%)	72.88 (16.80)	59.97-85.78	0.22	56.77 (14.68)	47.44-66.10	0.03
Iwama et al.^7^ (%)	79.94 (16.70)	67.09-92.76	0.22	62.24 (16.85)	51.53-72.95	0.02
Gibbons et al.^6^ (%)	66.53 (14.04)	55.72-77.31	0.22^a^	51.82 (13.87)	43.01-60.63	0.09^^a^^
Enright and Sherrill^5^ (%)	83.74 (17.85)	70.01-77.31	0.22	68.61 (20.01)	55.90-81.32	0.04

6MWT=six-minute walk test; PPC=postoperative pulmonary complications; SD=standard deviation; a: Gibbons ? all other equations (p=0.02); Britto A and B: equations without and with heart rate, respectively; pred=predicted.

The evaluation of risk factors for the development of PPC was significant for FEV_1_ and 6MWT, with an OR of 0.94 for FEV_1_ (p=0.04), while for the performance in the 6MWT, the OR was 22 (p=0.01). Other factors, such as age, sex, and BMI did not significantly correlate with the appearance of PPC (Table 3).

**Table 3:** - Risk factors and development of PPC.

Effects	Odds Ratio	CI95%	P value
Without PPC vs. with PPC	2.667	0.277-25.635	0.40
FEV_1_ (%)	0.94	0.89-0.99	0.04
6MWT (m)	22.00	1.86-260.65	0.01
Age	1.04	0.93-1.17	0.52
Gender (F x M)	0.36	0.06-2.16	0.26
BMI	1.04	0.86-1.27	0.69

PPC=postoperative pulmonary complications; FEV1=forced expiratory volume in one second; 6MWT=six-minute walk test; F=female; M=male.

## Discussion

In this study, the functional performance of 21 patients was evaluated using the 6MWT in the preoperative phase of open thoracotomy. In the postoperative follow-up, the development of PPC and performance in the 6MWT divided the volunteers into two groups. The first group included those with the best performances during the 6MWT who did not develop PPC. The second group included those who had worse performances, walked a shorter distance than that provided by the reference equations, and went on to develop PPC.

Although FEV_1_ has also been linked to the PPC, the chance of developing PPC increased as the 6MWD decreased, and these data suggest that the 6MWT, which is easy and simple to execute, can help the physical therapist and the team in planning the postoperative care routine, with more intense care in the group with a lower than expected performance in the 6MWT.

It is important to note that this study did not interfere in the care of these patients, who received the routine clinical care and physical therapy during their hospital stay, and the group that developed PPC comprised 57.14% of the study participants. The definition of PPC differs among authors, but can include an incidence of up to 65% of atelectasis and 20% pneumonia^12^. This rate may be associated with the quality of postoperative care. One study found a low incidence (7.7%) of PPC after lung resection performed by a trained multidisciplinary team^13^.

Although spirometry, using FEV_1_ and DLCO in the lung, have been suggested since the 1950s as good predictors of PPC, some studies have shown a good correlation between FEV_1_ and CPP^2^^,^ 14 ^-^ 17. In the study of Stanzani et al.^3^, lower FEV_1_ values predicted for the postoperative outcome correlated with a higher rate of PPC, although the cardiopulmonary exercise test has been important in identifying patients at high risk. In this context, it would be interesting to identify the 6MWT as a possible substitute for cardiopulmonary exercise testing, mainly due to its lower cost and simplicity.

In this study, the 6MWT and FEV_1_ variables correlated with an increased chance of PPC, especially the 6MWT. Subjects who walked way below the predicted distance in the 6MWT showed an increased chance of developing PPC, with an odds ratio of 22, while the decrease in FEV_1_ was related to increased PPC with an odds ratio of approximately one. These results suggest that the 6MWT was a better indicator of PPC risk in lung surgery, even considering the large amplitude of the resulting confidence interval.

PPCs are associated with increased hospital costs and greater exposure of patients to infections, in addition to the physical, emotional, and social burden. Preventing the onset of PPC requires interventions and more complex and/or frequent care, especially with regard to physical therapy services, and in this context the inclusion of the 6MWT in the preoperative evaluation could guide physical therapists and all clinical staff regarding which patients are likely to require more intensive or less intensive treatment. However, other studies found no relationship between the risk of developing PPC and performance in the incremental shuttle walk test or the endurance shuttle walk test in pulmonary surgery^2^ or between the 6MWT and PPC in abdominal surgery^18^.

The 6MWT is widely used in the literature for different purposes, such as measuring response to interventions in patients with heart and pulmonary diseases^4^. A systematic review of the walk test found that the 6MWT was most commonly used in patients with chronic obstructive pulmonary disease (COPD) or heart failure and, less frequently, in cases of surgery^19^. In these studies, the distance traveled was strongly correlated with maximal oxygen uptake (VO_2_ max) measured on a bike or treadmill. Some researchers have shown that reduced exercise capacity is associated with a longer hospital stay and costs in patients undergoing lung resection^20^^-^ 22. Other authors have shown that when the distance was 300 meters or less, the risk of hospitalization or mortality in patients with heart failure increased^23^. It is noteworthy that the average distance traveled by the group with PPC in our study was 340.89 meters.

The use of the 6MWT for preoperative evaluation involves the use of reference values for comparison between achieved performance and expected values for each individual. According to the guidelines of ATS^4^, the values for interpretation of the 6MWT should be based on sex, age, height, and weight of individuals, due to the interference of these variables on performance in the test. Even before the ATS standardization^4^ for the 6MWT, Enright and Sherrill^5^ published an equation that took into account the height, weight, age, and sex of individuals in relation to the 6MWT. Since then, with the popularization of the 6MWT, other equations for forecasting distance were proposed, and other variables were introduced.

In this study, it was necessary to test which one of the proposed equations to predict the 6MWT would be better suited to this population. Of the variables used in the equations, age, gender, weight and height, and BMI were the most frequent^5^^,^ 10 ^,^ 11. Britto et al.^11^ also proposed a second equation model with the change in heart rate between the start and end of the test, the latter equation having a greater affinity with the 6MWT, according to the authors. Soares and Pereira^8^ published an equation covering only the age, height, and BMI of the participants, while Gibbons et al.^6^ found only age and sex were relevant variables in their work, similarly to that found by Iwama et al.^7^, who worked out an equation composed of the variables age and sex.

As for the method used in the studies that resulted in the equations, Iwama et al.^7^, Soares and Pereira^8^, Dourado et al.^10^, and Britto et al.^11^, used the standardization of ATS^4^ and only Gibbons et al.^6^ used a hallway which was far less than 30 meters in length (20 meters used) and held the test three times.

The results showed that the group with PPC had an average walking distance of 340.89 meters, while the group without PPC traveled a greater distance, with an average of 422.38 meters. The performance of the group without PPC was approximately 80% of the values predicted by the equations analyzed in this study (except Gibbons et al.^6^), while the group with PPC averaged less 70% of the predicted values. Comparing the different equations used, proposed by Britto et al.^11^, Soares and Pereira^8^, Dourado et al.^10^, and Iwama et al.^7^, the predicted values showed no statistically significant difference, which may suggest that the latter are more appropriate for this population because they were able to predict the expected performance in the 6MWT. Among the equations developed in international research, the equation of Enright and Sherrill^5^ was the one with similar results to the Brazilian analyzed equations.

Importantly, the 6MWT requires activity at submaximal level, allowing for a greater variation of performance according to the effort employed by the patient, but the physiological variables were not examined during the test. Another limitation of the 6MWT can be attributed to the fact that equations available in the literature are not prepared based on a population such as that in this study, i.e. with prior disease and surgical indication.

In conclusion, the 6MWT is an easy, safe, and feasible test for routine preoperative evaluation in pulmonary surgery and may indicate patients with a higher chance of developing PPC. The reference equations for the Brazilian 6MWT of Britto et al.^11^, Soares and Pereira^8^, Dourado et al.^10^, and Iwama et al.^7^ seem more appropriate for this population, followed by the equation of Enright and Sherril^5^.

## References

[1] Silva DR, Baglio PT, Gazzana MB, Barreto SSM (2009). Avaliação pulmonar e prevenção das complicações respiratórias
perioperatórias. Rev Bras Clin Med.

[2] Erdoğan Y, Günay E, Ergün P, Kaymaz D, Temiz G, Karaoglanoglu N (2013). Can exercise capacity assessed by the shuttle walk test predict the
development of post-operative complications in patients with lung
cancer?. Tuberk Toraks.

[3] Stanzani F, Paisani DM, Oliveira A, Souza RC, Perfeito JAJ, Faresin SM (2014). Mortalidade, mortalidade e categorização de risco para complicações
perioperatórias em pacientes com câncer de pulmão. J Bras Pneumol.

[4] ATS Committee on Proficiency Standards for Clinical Pulmonary
Function Laboratories (2002). ATS statement: guidelines for the six-minute walk test. Am J Respir Crit Care Med.

[5] Enright PL, Sherrill DL (1998). Reference equations for the six-minute walk in healthy
adults. Am J Respir Crit Care Med.

[6] Gibbons WJ, Fruchter N, Sloan S, Levy RD (2001). Reference values for a multiple repetition 6-minute walk test in
healthy adults older than 20 years. J Cardiopulm Rehabil.

[7] Iwama AM, Andrade GN, Shima P, Tanni SE, Godoy I, Dourado VZ (2009). The six-minute walk test and body weight-walk distance product in
healthy Brazilian subjects. Braz J Med Biol Res.

[8] Soares MR, Pereira CA (2011). Six-minute walk test: reference values for healthy adults in
Brazil. J Bras Pneumol.

[9] Dourado VZ (2011). Reference equations for the 6-minute walk test in healthy
individuals. Arq Bras Cardiol.

[10] Dourado VZ, Vidotto MC, Guerra RL (2011). Reference equations for the performance of healthy adults on field
walking tests. J Bras Pneumol.

[11] Britto RR, Probst VS, Andrade AF, Samora GA, Hernandes NA, Marinho PE (2013). Reference equations for the six-minute walk distance based on a
Brazilian multicenter study. Braz J Phys Ther.

[12] Pasquina P, Tramèr MR, Granier JM, Walder B (2006). Respiratory physiotherapy to prevent pulmonary complications after
abdominal surgery: a systematic review. Chest.

[13] Reeve JC, Nicol K, Stiller K, McPherson KM, Birch P, Gordon IR (2010). Does physiotherapy reduce the incidence of postoperative pulmonary
complications following pulmonary resection via open thoracotomy? A preliminary
randomised single-blind clinical trial. Eur J Cardiothorac Surg.

[14] Pereira ED, Fernandes AL, Anção MS, Peres CA, Atallah AN, Faresin SM (1999). Prospective assessment of the risk of postoperative pulmonary
complications in patients submitted to upper abdominal surgery. Sao Paulo Med J.

[15] Mazzone PJ, Arroliga AC (2005). Lung cancer: preoperative pulmonary evaluation of the lung resection
candidate. Am J Med.

[16] Nosotti M, Baisi A, Mendogni P, Palleschi A, Tosi D, Rosso L (2010). Muscle sparing versus posterolateral thoracotomy for pulmonary
lobectomy: randomised controlled trial. Interact Cardiovasc Thorac Surg.

[17] Troosters T, Gosselink R, Decramer M (1999). Six-minute walking distance in healthy elderly
subjects. Eur Respir J.

[18] Paisani DM, Fiore JF Jr, Lunardi AC, Colluci DB, Santoro IL, Carvalho CR (2012). Preoperative 6-min walking distance does not predict pulmonary
complications in upper abdominal surgery. Respirology.

[19] Solway S, Brooks D, Lacasse Y, Thomas S (2001). A qualitative systematic overview of the measurement properties of
functional walk tests used in the cardiorespiratory domain. Chest.

[20] Pelletier C, Lapointe L, Leblanc P (1990). Effects of lung resection on pulmonary function and exercise
capacity. Thorax.

[21] Weinstein H, Bates AT, Spaltro BE, Thaler HT, Steingart RM (2007). Influence of preoperative exercise capacity on length of stay after
thoracic cancer surgery. Ann Thorac Surg.

[22] Brunelli A, Refai M, Xiumé F, Salati M, Sciarra V, Socci L (2008). Desempenho no teste de escada limitado por sintomas está associada com
aumento de complicações cardiopulmonares, mortalidade e custos após grande
ressecção pulmonar. Ann Thorac Surg.

[23] Pancieri MV, Cataneo DC, Montovani JC, Cataneo AJ (2010). Comparison between actual and predicted postoperative stair-climbing
test, walk test and spirometric values in patients undergoing lung
resection. Acta Cir Bras.

